# A Review of Stoichiometric Nickel Sulfide-Based Catalysts for Hydrogen Evolution Reaction in Alkaline Media

**DOI:** 10.3390/molecules29204975

**Published:** 2024-10-21

**Authors:** Yeji Choi, Jun-Hee Lee, Duck Hyun Youn

**Affiliations:** Department of Chemical Engineering, Department of Integrative Engineering for Hydrogen Safety, Kangwon National University, Chuncheon 24341, Republic of Korea; yeahd27@kangwon.ac.kr (Y.C.); jhlee16@kogas-tech.co.kr (J.-H.L.)

**Keywords:** nickel sulfides, hydrogen evolution reaction, stoichiometry, catalyst

## Abstract

Efficient and cost-effective catalysts for hydrogen evolution reaction (HER) are essential for large-scale hydrogen production, which is a critical step toward reducing carbon emissions and advancing the global transition to sustainable energy. Nickel sulfide-based catalysts, which exist in various stoichiometries, show promise for HER in alkaline media. However, as single-phase materials, they do not demonstrate superior activity compared to Pt-based catalysts. This review highlights recent strategies to enhance the HER performance of nickel sulfides, including heteroatom doping, heterostructure construction, and vacancy engineering, tailored to their different stoichiometric ratios. The study also examines synthesis methods, characterizations, and their impact on HER performance. Furthermore, it discusses the challenges and limitations of current research and suggests future directions for improvement.

## 1. Introduction

As climate and energy crises intensify, various agendas have been proposed to achieve net-zero emissions by 2050 [1,2]. In response, a transition to renewable energy, coupled with a reduction in fossil fuel consumption, is being proposed as a fundamental solution. To facilitate this energy transition, numerous policies and research initiatives have focused on renewable energy sources, such as solar, wind, hydro, geothermal, and hydrogen energy. Among these, hydrogen energy is gaining attention as a clean and sustainable renewable energy source due to its high gravimetric energy density, versatility, and zero emissions when used. These characteristics not only make it a valuable energy source but also enable it to serve as an energy carrier for storing excess power generated from other renewable sources. 

Despite these advantages, the environmental impact of hydrogen varies significantly depending on its production method. Hydrogen is classified by color based on production methods and is broadly categorized as gray, blue, and green hydrogen. Gray hydrogen is produced from fossil fuels, primarily by steam methane reforming (SMR) of liquefied natural gas. Blue hydrogen incorporates carbon capture and storage (CCS) technology into the gray hydrogen process to reduce carbon emissions; however, both methods still rely on fossil fuels and contribute to greenhouse gas emissions. In contrast, green hydrogen is produced using electricity to electrolyze water, generating hydrogen without harmful byproducts [3]. 

Water electrolysis involves anodic oxygen evolution reaction (OER) and cathodic hydrogen evolution reaction (HER). Although the theoretical potential for water electrolysis is 1.23 V, the actual potential required in an electrolyzer is significantly higher because of overpotentials at both the anode and cathode. Particularly in HER, precious Pt-based catalysts are typically used, but their high cost and limited availability hinder the widespread adoption of water electrolysis systems. Therefore, developing efficient and cost-effective electrocatalysts for HER is crucial. 

**Figure 1:**
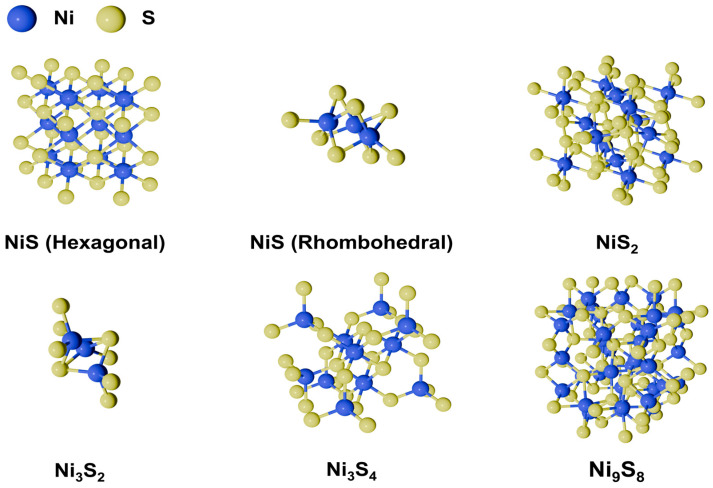
Primitive cell structure of nickel sulfides as a HER catalyst.

Recently, various catalysts of transition metal-based compounds, such as nitrides [4,5,6], carbides [7,8,9], phosphides [10,11,12], and sulfides [13,14,15,16,17,18], have been extensively studied as non-precious HER catalysts. Among these materials, nickel sulfide stands out due to its cost-effectiveness, natural abundance, excellent electrical conductivity, and stability in alkaline environments, where it remains insoluble within electrolytes [19]. These properties make nickel sulfide a promising replacement for traditional Pt-based catalysts. Notably, nickel sulfide exists in various stoichiometries and has been reported as a HER catalyst in several forms, including hexagonal NiS, rhombohedral NiS, cubic NiS_2_, trigonal Ni_3_S_2_, cubic Ni_3_S_4_, and tetragonal Ni_9_S_8_ (Figure 1). However, when used alone, nickel sulfide exhibited significantly lower activity and stability for HER compared to Pt-based catalysts. To address this limitation, numerous studies have explored strategies to enhance HER performance, such as heteroatom doping, the construction of nickel sulfide-based heterostructures, and vacancy engineering. These approaches aim at optimizing the electronic structure and surface properties of nickel sulfide to improve HER performance.

In this review, we summarize and categorize research findings on enhancing the performance of nickel sulfide-based catalysts, focusing on their synthesis methods, characterizations, and HER performance according to their stoichiometries. The limitations and potential improvements of these catalysts are also discussed. 

## 2. Mechanisms of HER in Alkaline Media

The overall water splitting reaction occurring in the electrolysis is as follows, involving a two-electron transfer reaction:

H_2_O → H_2_ + 1/2 O_2_ (1)

**Figure 2:**
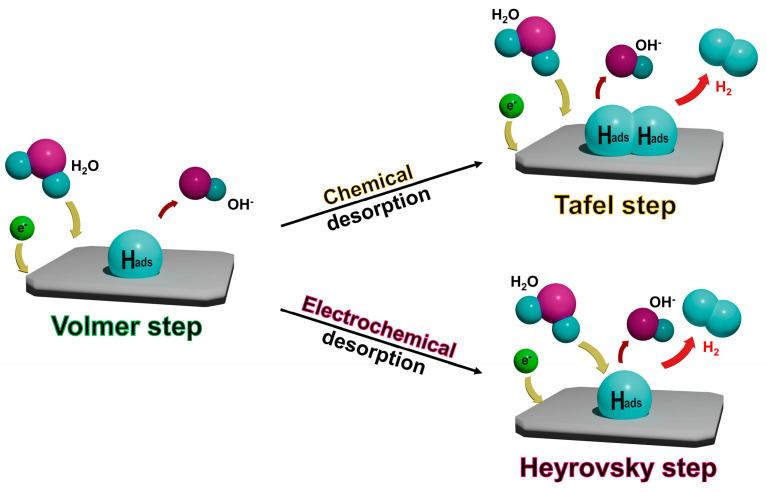
Schematic illustration of the HER mechanisms in alkaline media.

In an alkaline environment, the HER occurs via two mechanisms at the cathode (Figure 2). Initially, water molecules react with electrons, leading to the dissociation of water into adsorbed hydrogen atoms (denoted as H_ads_) and hydroxide ions (OH^−^). This initial reaction is known as the Volmer step:

H_2_O + e^−^ → H_ads_ + OH^−^ (2)

Following the Volmer step, the H_ads_ can follow two distinct pathways to form hydrogen gas (H_2_). The first pathway is the Tafel step, where two H_ads_ atoms on the catalyst surface combine chemically to produce molecular hydrogen (H_2_):

H_ads_ + H_ads_ → H_2_ (3)

In contrast, the second pathway is the Heyrovsky step, which involves an electrochemical process. In this pathway, a H_ads_ reacts with a water molecule and an additional electron, producing hydrogen gas (H_2_) and a hydroxide ion (OH^−^):

H_ads_ + H_2_O + e^−^ → H_2_ + OH^−^ (4)

The determination of the Tafel and Heyrovsky pathways depends on the desorption method of H_ads_. If H_ads_ is released through chemical desorption, it follows the Tafel step, whereas if the release involves electrochemical desorption, it proceeds through the Heyrovsky step. These two steps occur simultaneously during the HER process, and depending on which step is dominant, the mechanism is referred to as either the Volmer–Tafel mechanism or the Volmer–Heyrovsky mechanism. 

## 3. Stoichiometric Nickel Sulfide-Based Catalysts for HER 

### 3.1. NiS-Based Catalysts

#### 3.1.1. Hexagonal NiS-Based Catalysts

**Figure 3:**
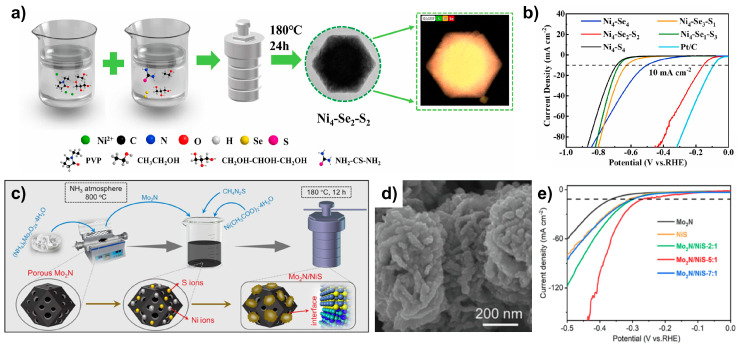
(**a**) Schematic illustration of the synthesis of NiS/NiSe_2_. (**b**) Linear sweep voltammetry (LSV) curves of Ni_x_–Se_y_–S_z_ in 1.0 M KOH. (**c**) Schematic representation of the synthesis of Mo_2_N/NiS. (**d**) SEM image showing the morphology of Mo_2_N/NiS. (**e**) LSV curves comparing Mo_2_N/NiS with its comparison group in 1.0 M KOH. Reproduced with permission from Elsevier [20,21].

Li et al. synthesized NiS/NiSe_2_ via a one-step hydrothermal method (Figure 3a) [20]. Scanning electron microscopy (SEM) images confirmed the formation of a polyhedral shape, while energy dispersive X-ray spectroscopy (EDS) elemental mapping showed that Ni and Se were uniformly distributed within the nanoparticles, with S concentrated in the inner regions. Adjusting the concentrations of S and Se during synthesis (Ni_x_–Se_y_–S_z_, where x, y, and z denote mmol of each element) revealed that an increase in S concentration (S_z_) led to irregular morphologies and smaller particle sizes. Controlling the morphology through concentration adjustments significantly affected the HER activity. NiS/NiSe_2_ (denoted as Ni_4_–Se_2_–S_2_), with its uniform polyhedral shape, exhibited a low overpotential of 155 mV at 10 mA cm^−2^(η_10_) in 1.0 M KOH (Figure 3b). This heterostructure demonstrated superior HER performance compared to NiS (Ni_4_–S_4_, η_10_ = 689 mV) and NiSe_2_ (Ni_4_–Se_4_, η_10_ = 502 mV).

Mo_2_N/NiS was prepared by annealing followed by hydrothermal treatment (Figure 3c) [21]. Initially, ammonium molybdate was decomposed via annealing under an NH_3_ atmosphere to afford a porous Mo_2_N substrate. Subsequent hydrothermal treatment with Ni and S sources yielded the Mo_2_N/NiS heterostructure. The SEM images confirmed that the hydrothermal treatment resulted in NiS particles anchored to the porous Mo_2_N structure (Figure 3d). Due to the formation of the heterostructure, Mo_2_N/NiS exhibited improved activity in 1.0 M KOH, with an overpotential η_10_ = 254 mV, compared with Mo_2_N (η_10_ = 355 mV) and NiS (η_10_ = 300 mV) (Figure 3e). To understand the enhanced HER activity resulting from heterostructure formation, density functional theory (DFT) calculations were conducted. The model shows that Mo_2_N/NiS is connected by Mo–S bonds within the heterostructure, with electron accumulation at the S sites, consistent with X-ray photoelectron spectroscopy (XPS) results. Additionally, when the HER occurs, H_2_O first adsorbs on the Mo-site of Mo_2_N, followed by proton adsorption on the S site of NiS. Subsequently, OH^−^ desorbs, leading to the formation of H* and the desorption of H_2_. The calculated Gibbs free energy of the HER process indicated that water dissociation occurred more readily on NiS than on Mo_2_N, whereas stronger H* adsorption on NiS minimized the HER activity. However, when the Mo_2_N/NiS heterostructure was formed, water dissociation was facilitated, and the H* adsorption energy was optimized, leading to enhanced HER activity. 

Shi et al. synthesized a nitrogen-doped carbon (NC) coated NiS–CeO_2_ directly on Ni foam using a hydrothermal method followed by annealing [22]. SEM images revealed that nanorod structures formed during the initial hydrothermal process. After annealing with thiourea, a coated morphology was observed on the existing nanorods. This coating layer was confirmed by Fourier transform infrared spectroscopy (FT-IR), which identified a C–S adsorption peak, and XPS analysis of the C 1s and N 1s spectra confirmed the presence N-doped carbon on the nanorods. The authors suggested that NC layer enhances conductivity and stability in alkaline media. The NC/NiS–CeO_2_ catalyst demonstrated excellent HER activity with overpotential of η_10_ = 47 mV and η_50_ = 108 mV, outperforming Ni–CeO_2_ (η_10_ = 207 mV and η_50_ = 352 mV) before the formation of the NiS and NC layer. Furthermore, the introduction of CeO_2_ improved activity compared to NC/NiS alone (η_10_ = 122 mV and η_50_ = 219 mV).

MoS_2_/NiS catalyst was synthesized using a one-step hydrothermal method, resulting in a 3D needle-like morphology directly grown on Ni foam (NF), as observed in SEM images [23]. High-resolution SEM confirmed that the framework was composed of 2D nanosheets, and transmission electron microscopy (TEM) images showed MoS_2_/NF was coated on the needle-like hierarchical nanostructure. This unique morphology contributed to the superior HER performance of MoS_2_/NiS/NF, which exhibited a low overpotential of η_10_ = 87 mV in 1.0 M KOH, surpassing those of the single components NiS (η_10_ = 191 mV) and MoS_2_ (η_10_ = 145 mV). Furthermore, the MoS_2_/NiS/NF exhibited nearly twice the double-layer capacitance (C_dl_) compared to individual components (NiS/NF: 2.38 mF cm^−2^, MoS_2_/NF: 14.2 mF cm^−2^), with MoS_2_/NiS/NF showing 27.6 mF cm^−2^, indicating a significantly enhanced electrochemical active surface area. The HER performance of recently reported hexagonal NiS-based HER catalysts are summarized in Table 1. 

#### 3.1.2. Rhombohedral NiS-Based Catalysts

**Figure 4:**
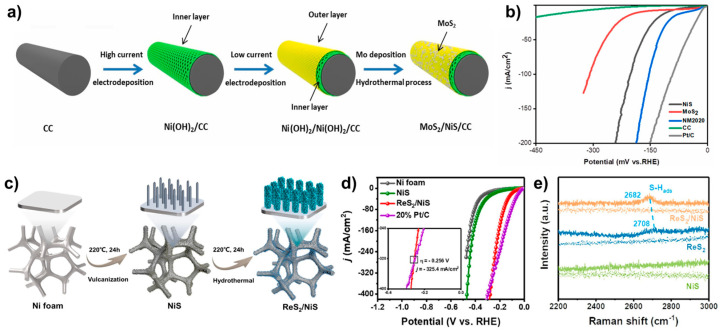
(**a**) Schematic illustration of the synthesis of MoS_2_/NiS/CC. (**b**) LSV curves comparing MoS_2_/NiS/CC (denoted as NM2020) with its control group in 1.0 M KOH. (**c**) Schematic representation of the synthesis of ReS_2_/NiS. (**d**) LSV curves comparing ReS_2_/NiS with its control group in 1.0 M KOH. (**e**) Raman spectra of NiS, ReS_2_ and ReS_2_/NiS before (dotted) and after (solid) stability tests. Reproduced with permission from Elsevier [28,29].

Shi et al. synthesized MoS_2_/NiS using a two-step electrodeposition process followed by a hydrothermal treatment (Figure 4a) [28]. Initially, a two-step electrodeposition formed layered Ni(OH)_2_–Ni(OH)_2_/carbon cloth (CC) structures. MoS_2_ was then generated via hydrothermal treatment of the Ni(OH)_2_/CC, resulting in MoS_2_/NiS/CC. Compared with NiS/CC (η_20_ = 134 mV) and MoS_2_/CC (η_20_ = 224 mV), MoS_2_/NiS (NM2020, where 20 denotes the applied current density during each electrodeposition step) demonstrated significantly enhanced HER activity, achieving an η_20_ of 97 mV (Figure 4b). The applied current density variations influenced the electrodeposition rate and consequently the crystal core size. Applying a high current density (50 mA cm^−2^) during the first step and a low current density (30 mA cm^−2^) in the second step resulted in the formation of loose networks in the inner layer and dense structures in the outer layer. This configuration resulted in the synthesized catalyst (NM5030) exhibiting the highest HER activity, with η_10_ = 18 mV and η_100_ = 93 mV, attributed to the lattice strain induced by the structural differences between the two layers.

ReS_2_/NiS catalyst was prepared using a two-step hydrothermal process (Figure 4c) [29]. Initially, NiS nanowires were formed on the Ni foam via vulcanization. In the second step, these nanowires were combined with a Re precursor to form ReS_2_/NiS, where ReS_2_ nanosheets was deposited onto the NiS nanowires. This unique morphology resulted in ReS_2_/NiS exhibiting an overpotential of η_10_ = 78 mV in 1.0 M KOH, demonstrating superior HER activity to the single-phase of NiS nanowires (Figure 4d). Notably, at current densities above 325 mA cm^−2^, the ReS_2_/NiS catalyst outperformed the Pt/C catalyst. In-situ Raman spectroscopy was employed to investigate the enhanced HER activity by comparing fresh samples with those subjected to the HER reaction at 150 mA cm^−2^ for 10 h (denoted as “tested samples”). For NiS, no bands were observed in the tested samples; however, for ReS_2_ and ReS_2_/NiS, an S–H_ads_ band appeared at 2708 cm^−1^ and 2682 cm^−1^, respectively (Figure 4e). The red shift in the S–H_ads_ band of ReS_2_/NiS indicates that the S–H_ads_ bond is weaker than in the single phase of ReS_2_. Consequently, the formation of a heterostructure creates a more favorable surface state for HER. Work function derived from DFT calculations revealed that the Fermi level of NiS is higher than that of ReS_2_, indicating that a Schottky junction is created when these two materials form a heterojunction. This drives electron transfer from NiS to ReS_2_, consistent with the XPS results. During the HER in the single-phase NiS and ReS_2_, OH^−^ favored adsorption onto the Ni and Re sites, respectively, while H transferred to the S sites. However, NiS exhibited weak water adsorption, and ReS_2_ formed a strong S-H_ads_ bond, both of which hindered HER activity. In contrast, in the NiS/ReS_2_ heterostructure, electron transfer from NiS to ReS_2_ resulted in OH^−^ adsorption on the Ni site of NiS and H^+^ adsorption on the S site of ReS_2_, resulting in a more suitable energy state for HER. 

Huang et al. directly synthesized NiS/MoS_2_ on calcined carbon paper (CP) using a one-step hydrothermal method [30]. The calcination process altered the hydrophobic/ aerophilic surface of CP to hydrophilic/aerophobic, as confirmed by contact angle measurements. This transformation enhanced the interaction between water and the electrode during the HER, facilitating the detachment of the generated gas from the electrode surface. Consequently, NiS/MoS_2_ on the calcined CP demonstrated notable HER performance with a η_10_ = 119 mV in 1.0 M KOH, outperforming the individual components NiS/CP (η_10_ = 225 mV) and MoS_2_/CP (η_10_ = 127 mV).

Jiang et al. synthesized a MoS_2_/NiS core-shell structure via a one-step hydrothermal method [31]. SEM images revealed that MoS_2_/NiS formed nanorods on nickel foam, in contrast to the nanoparticle morphology of MoS_2_ and the nanosheet structure form of NiS. TEM images revealed that MoS_2_/NiS had fluffy surfaces with both MoS_2_ and NiS coexisting. After removing the surface components by ultrasonication, only NiS remained, indicating that NiS formed the backbone with MoS_2_ coating the surface. This morphological effect resulted in excellent HER activity in 1.0 M KOH, achieving η_10_ = 84 mV, which significantly improved upon the lower activity of NiS alone (η_10_ = 176 mV) due to the heterostructure formation with MoS_2_. The HER performance of recently reported rhombohedral NiS-based HER catalysts is summarized in Table 2. 

### 3.2. NiS_2_-Based Catalysts

Liu et al. synthesized Co–doped CeO_2_/NiS_2_ on Ni foam (Co–CeO_2_/NiS_2_/NF) using a hydrothermal method followed by annealing [34]. During the hydrothermal step, Ce, Ni, and Co precursors formed an intermediate, which was then vulcanized under an Ar atmosphere. SEM images revealed that Co–CeO_2_/NiS_2_/NF exhibited a nanoflower-like morphology with nanosheets directly formed on Ni foam, providing a larger specific surface area. The double-layer capacitance (C_dl_) value increased significantly with the introduction of Co doping (Co–NiS_2_/NF, 62.61 mF cm^−2^) or the formation of a heterostructure with CeO_2_ (NiS_2_/CeO_2_/NF, 61.92 mF cm^−2^) compared to single-phase NiS_2_/NF (25.37 mF cm^−2^). The highest C_dl_ value of 83.52 mF cm^−2^ was observed in the Co–NiS_2_/CeO_2_/NF, demonstrating that Co doping and heterostructure formation with CeO_2_ in NiS_2_ significantly enhances the electrochemical active surface area. The Co–CeO_2_/NiS_2_/NF catalyst exhibited improved HER performance in 1.0 M KOH, achieving an η_10_ value of 84 mV, outperforming single NiS_2_/NF (η_10_ = 149 mV), the catalyst without Co doping, NiS_2_/CeO_2_/NF (η_10_ = 108 mV), and the catalyst without heterostructure, Co–NiS_2_/NF (η_10_ = 109 mV).

**Figure 5:**
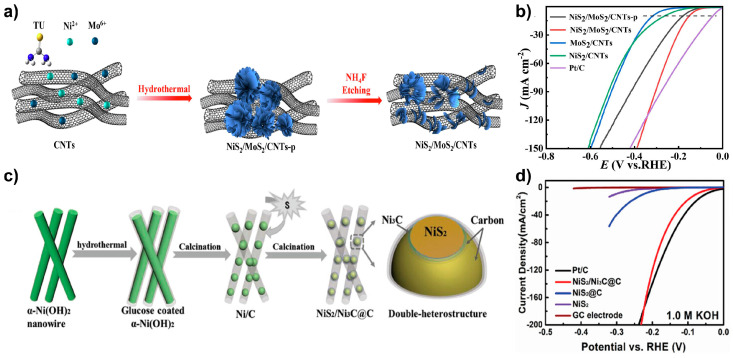
(**a**) Schematic illustration of the synthesis of NiS_2_/MoS_2_/CNTs. (**b**) LSV curves comparing NiS_2_/MoS_2_/CNTs with control group in 1.0 M KOH. (**c**) Schematic representation of the synthesis of NiS_2_/Ni_3_C@C. (**d**) LSV curves comparing NiS_2_/Ni_3_C@C with its control samples in 1.0 M KOH. Reproduced with permission from Elsevier [35] and Wiley [36].

Li et al. synthesized NiS_2_/MoS_2_/CNTs using a hydrothermal process followed by etching process in NH_4_F solution (Figure 5a) [35]. SEM images showed that NiS_2_/MoS_2_/CNTs exhibited a flower-like morphology on the CNT surface, unlike MoS_2_/CNTs and NiS_2_/CNTs. After the etching process, the nanospheres were partially destroyed, indicating the partial removal of the NiS_2_/MoS_2_. Raman spectroscopy detected Mo_3_S_13_ edge sites at 819, 889, and 937 cm^−1^ were detected after the etching process, indicating the formation of abundant Mo–S edge sites. These Mo–S edge sites have insufficient coordination compared to the basal plane, facilitating easier hydrogen adsorption and desorption. The etching process significantly enhanced the HER activity, reducing the overpotential from 178 to 149 mV at 10 mA cm^−2^ in 1.0 M KOH. This heterostructure exhibited superior performance compared to single-phase MoS_2_ (η_10_ = 316 mV) and NiS_2_ (η_10_ = 260 mV), demonstrating enhanced catalytic activity (Figure 5b). 

NiS_2_/Ni_3_C@C catalyst was prepared via a two-step hydrothermal process followed by a two-step calcination process (Figure 5c) [36]. Initially, α-Ni(OH)_2_ was synthesized via hydrothermal treatment. Glucose was then added as a carbon source, followed by an additional hydrothermal treatment to coat the catalyst with carbon. The catalyst underwent calcination at 700 °C, followed by a second calcination at 450 °C using thiourea as the sulfur source, forming NiS_2_/Ni_3_C@C. SEM images revealed that NiS_2_/Ni_3_C@C exhibited a peapod-like morphology with nanoparticles embedded within amorphous carbon, while TEM images indicated that Ni_3_C encapsulated the NiS_2_ nanoparticles. NiS_2_/Ni_3_C@C demonstrated an η_10_ value of 78 mV in 1.0 M KOH, which was significantly lower than that of NiS_2_@C (η_10_ = 226 mV) (Figure 5d). Furthermore, at current densities exceeding 200 mA cm^−2^, NiS_2_/Ni_3_C@C showed superior activity compared to Pt/C. DFT calculations revealed that the density of states (DOS) of Ni_3_C exhibits a metal-like structure, whereas defected carbon displays a semiconductor-like structure, suggesting the formation of a Schottky barrier at the Ni_3_C/C interface. This configuration facilitates faster electron and proton diffusion, adjusting the high Gibbs free energy of hydrogen adsorption in NiS_2_ to near zero through heterostructure formation. 

Vanadium-doped NiS_2_ on carbon cloth (V–NiS_2_/CC) catalyst was synthesized via a hydrothermal method followed by annealing [37]. For the single NiS_2_/CC catalyst, SEM images showed that NiS_2_ aggregated on the carbon cloth. However, V doping, resulted in vertically grown nanosheets on the carbon cloth, indicating that V introduction promotes the formation of a morphology with a larger surface area. As a result, V–NiS_2_/CC exhibited a lower η_10_ value of 85 mV than NiS_2_/CC (η_10_ = 115 mV). DFT calculations indicated that V doping alters the electronic structure of NiS_2_ from semiconductor to metallic, enhancing its electrical conductivity. Additionally, V doping significantly reduced the water dissociation energy barrier, thereby lowering the overpotential. The HER performance of recently reported NiS_2_-based HER catalysts is summarized in Table 3.

### 3.3. Ni_3_S_2_-Based Catalysts

Tang et al. synthesized Co–MoS_2_/Ni_3_S_2_/NF via a one-step hydrothermal process [46]. SEM images showed that, both Co–MoS_2_/Ni_3_S_2_/NF and undoped MoS_2_/Ni_3_S_2_/NF formed flower-like aggregates, irrespective of Co introduction. However, XPS analysis identified doublets in the Mo^4+^ peak, indicating the co-existence of both 1T–MoS_2_ and 2H–MoS_2_ phases in Co–MoS_2_/Ni_3_S_2_/NF and MoS_2_/Ni_3_S_2_/NF. Notably, Co doping significantly increased the proportion of the 1T–MoS_2_ phase, suggesting that Co promotes the phase transition from 2H–MoS_2_ to 1T–MoS_2_. The increased 1T–MoS_2_, content enabled Co–MoS_2_/Ni_3_S_2_/NF to achieve remarkable HER performance, with η_10_ and η_100_ values of 43 and 201 mV, respectively, in 1.0 M KOH. This represents a significant improvement compared to MoS_2_/Ni_3_S_2_/NF (η_10_ = 107 mV and η_100_ = 355 mV) and surpasses the activity of Ni_3_S_2_/NF (η_10_ = 245 mV and η_100_ = 494 mV), highlighting the advantages of heterostructure formation. 

**Figure 6:**
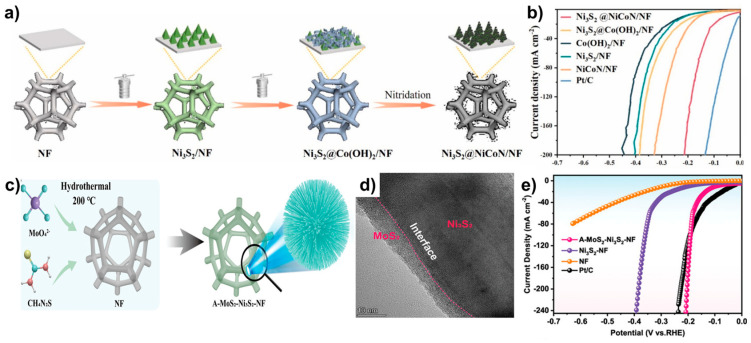
(**a**) Schematic illustration of the synthesis of Ni_3_S_2_@NiCoN/NF. (**b**) LSV curves comparing of Ni_3_S_2_@NiCoN/NF with control groups in 1.0 M KOH. (**c**) Schematic representation of the synthesis of A–MoS_2_–Ni_3_S_2_–NF. (**d**) HRTEM image of A-MoS_2_-Ni_3_S_2_-NF. (**e**) LSV curves comparing of A–MoS_2_–Ni_3_S_2_–NF with control samples in 1.0 M KOH. Reproduced with permission from Elsevier [47] and Wiley [48].

Ni_3_S_2_–Ni_3_N–Co_2_N_0.67_/NF (Ni_3_S_2_@NiCoN/NF) catalyst was prepared via a two-step hydrothermal process followed by annealing (Figure 6a) [47]. Initially, Ni_3_S_2_/NF was synthesized via conducting hydrothermal treatment of Ni foam with thiourea. Cobalt chloride introduced in a subsequent hydrothermal step to form Ni_3_S_2_@Co(OH)_2_. Finally, the catalyst was annealed under an ammonia (NH_3_) flow, serving as a nitrogen source, to synthesize Ni_3_S_2_@NiCoN/NF. SEM images revealed that the introduction of Ni_3_N and Co_2_N_0.67_ into Ni_3_S_2_/NF, transformed the original cone structure of Ni_3_S_2_/NF into a core–shell triangular cone structure, which exposed more active sites. This structural transformation led to Ni_3_S_2_@NiCoN/NF demonstrating superior HER activity compared to Ni_3_S_2_/NF (η_10_ = 215 mV), with η_10_, η_50_ and η_100_ values of 63, 141 and 174 mV, respectively in 1.0 M KOH (Figure 6b).

Hu et al. synthesized Ni_3_S_2_ decorated with amorphous MoS_2_ (A–MoS_2_–Ni_3_S_2_–NF) using a one-step hydrothermal method (Figure 6c) [48]. SEM images revealed that A–MoS_2_–Ni_3_S_2_ formed an urchin-like morphology composed of assembled nanorods, and SEM-EDS measurements confirmed the presence of amorphous MoS_2_ based on the observed atomic ratios. TEM images further demonstrated that Ni_3_S_2_ formed the core, with amorphous MoS_2_ acting as the shell (Figure 6d). XPS spectra indicate electron transfer from inner Ni_3_S_2_ to outer amorphous MoS_2_ in the core–shell structure. Additionally, the introduction of amorphous MoS_2_ likely induced defects on the basal plane, maximizing the exposure of active sites. Consequently, A–MoS_2_–Ni_3_S_2_–NF exhibited outstanding HER performance, with η_10_ of 95 mV and η_100_ of 191 mV in 1.0 M KOH, significantly surpassing single Ni_3_S_2_–NF (η_10_ = 198 mV, η_100_ = 359 mV) (Figure 6e). The superior activity of A–MoS_2_–Ni_3_S_2_–NF can be attributed to the electron-rich environment of amorphous MoS_2_ on the catalyst’s surface, which promotes water dissociation and facilitates the Volmer and Heyrovsky process through rapid electron transfer reactions.

Li, V co-doped Ni_3_S_2_ (Li, V–Ni_3_S_2_) catalysts were prepared via a one-step hydrothermal process [49]. SEM images revealed that undoped Ni_3_S_2_ formed a granular shape on the Ni foam, whereas V doping yielded a well-arranged nanorod array. With Li and V co-doping, the nanorods developed a rougher surface compared to V–doped Ni_3_S_2_, suggesting enhanced provision of active sites, thereby facilitating electron and mass transfer. The Li, V–Ni_3_S_2_ exhibited excellent HER activity, with η_10_ of 90 mV and η_100_ of 183 mV in 1.0 M KOH, outperforming single Ni_3_S_2_ (η_10_ = 167 mV and η_100_ = 324 mV). Notably, a 2 × 2 cm^2^ single cell using Li, V–Ni_3_S_2_ as both the cathode and anode was employed for overall water splitting. It demonstrated exceptional activity at high current densities of 500 and 1000 mA cm^−2^, with cell potentials of 1.92 and 2.02 V, respectively. The single cell remained stable for 200 h under operation at 1000 mA cm^−2^.

**Figure 7:**
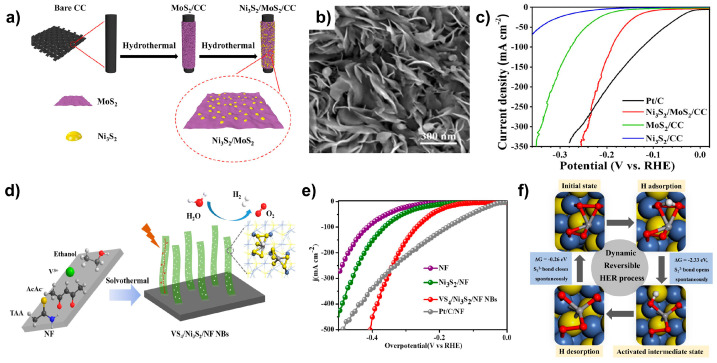
(**a**) Schematic illustration of the synthesis of Ni_3_S_2_/MoS_2_/CC. (**b**) SEM image showing the morphology of Ni_3_S_2_/MoS_2_/CC. (**c**) LSV curves comparing of Ni_3_S_2_/MoS_2_/CC with control samples in 1.0 M KOH. (**d**) Schematic representation of the synthesis of VS_4_/Ni_3_S_2_/NF NBs. (**e**) LSV curves comparing of VS_4_/Ni_3_S_2_/NF NBs with control groups in 1.0 M KOH. (**f**) Schematic diagram illustrating the reversible HER process of VS_4_/Ni_3_S_2_. Reproduced with permission from Elsevier [50,51].

Zhang et al. synthesized Ni_3_S_2_/MoS_2_ via a two-step hydrothermal process (Figure 7a) [50]. MoS_2_ was first formed on the carbon cloth (CC) followed by the growth of Ni_3_S_2_ on the MoS_2_. SEM images revealed the formation of Ni_3_S_2_/CC nanoparticles ranging in size from 6 to 22 nm on the MoS_2_ nanosheets (Figure 7b). After incorporating Ni_3_S_2_ into MoS_2_, X-ray diffraction (XRD) patterns showed a negative shift in the MoS_2_ (002) peak. Additionally, the HRTEM images of Ni_3_S_2_/MoS_2_/CC indicated that the layer spacing of MoS_2_ increased with more discontinuous lattice fringes compared to MoS_2_ alone. These findings suggest that Ni_3_S_2_ incorporation introduced defects and exposed more active sites on MoS_2_, enhancing its activity. Ni_3_S_2_/MoS_2_/CC exhibited superior activity compared to MoS_2_/CC and Ni_3_S_2_/CC, achieving an excellent performance of 189.4 mV at 100 mA cm^−2^ in 1.0 M KOH (Figure 7c) and outperforming Pt/C at current densities above 261.7 mA cm^−2^. Tafel slope reveals that the rate-determining step (RDS) for Ni_3_S_2_/CC (140.94 mVdec^−1^), MoS_2_ (97.34 mVdec^−1^), Ni_3_S_2_/MoS_2_/CC(81.01 mVdec^−1^) is the Volmer step. The formation of the heterostructure results in small Ni_3_S_2_ particles being present on MoS_2_, where Ni_3_S_2_, which effectively attracts oxygen-containing groups, adsorbs OH^−^, while MoS_2_ strongly binds to protons. This synergistic effect promotes the dissociation of water, leading to a reduction in the Tafel slope.

Yang et al. synthesized VS_4_/Ni_3_S_2_/NF via a one-step solvothermal process using vanadium (III) acetylacetonate and thioacetamide as the vanadium and sulfur sources, respectively (Figure 7d) [51]. SEM and TEM images confirmed that VS_4_ nanoparticles decorate the Ni_3_S_2_ nanobelt array grown on the Ni foam. To investigate the impact of the vanadium source on morphology, sodium metavanadate was used instead of vanadium acetylacetonate, resulting in the formation of nanorod arrays, highlighting the role of vanadium acetylacetonate in forming the nanobelt structure. The VS_4_/Ni_3_S_2_/NF exhibited significantly enhanced HER activity compared to Ni_3_S_2_/NF (η_10_ = 195 mV and η_100_ = 365 mV), achieving η_10_ of 140 mV and η_100_ of 268 mV in 1.0 M KOH (Figure 7e). DFT calculations indicated that the S–S bonds in VS_4_/Ni_3_S_2_ are crucial for enhancing HER activity (Figure 7f). During the HER process, H_2_O initially bonds to the S–S bonds, forming S–H_ads_. The bidentate S-S bond spontaneously opens, accumulating electrons around the S atom that are then transferred to H_ads_, enhancing HER activity. Subsequently, H_ads_ is released as H_2_, and the opened bridge spontaneously closes, returning to its initial state. This reversible process is repeated, supporting sustained HER performance. 

Co_2_P–Ni_3_S_2_/NF catalyst was fabricated through a two-step heat treatment process (hydrothermal followed by annealing) [52]. Initially, Co–Ni_3_S_2_/NF was synthesized via a hydrothermal reaction and then annealed under an N_2_ atmosphere with a phosphorus source to form Co_2_P–Ni_3_S_2_/NF. SEM images revealed that Co_2_P–Ni_3_S_2_/NF formed a uniform nanowire structure on the Ni foam, with the phosphating process resulting in thinner and finer nanowires. Co_2_P–Ni_3_S_2_/NF exhibited low overpotential at high current densities and superior activity compared to P–Ni_3_S_2_/NF, achieving η_100_ = 110 mV, η_500_ = 164 mV, η_1000_ = 196 mV in 1.0 M KOH, while P–Ni_3_S_2_ showed η_100_ = 262 mV and η_500_ = 385 mV. DFT calculations suggested that single Ni_3_S_2_ and Co_2_P have strong binding energies for H*, while Co_2_P–Ni_3_S_2_ exhibited near-zero Gibbs free energy of H*, enhancing the HER process.

In studies comparing NiS, NiS_2_, and Ni_3_S_2_, it has been reported that Ni_3_S_2_ exhibits relatively high activity [53,54]. The shorter bond lengths between Ni-S and Ni-Ni in Ni_3_S_2_ lead to stronger metal–metal bonding interactions. Furthermore, DOS calculations have shown that Ni_3_S_2_ possesses stronger metallic properties compared to NiS and NiS_2_, resulting in enhanced electrical conductivity and improved HER activity. As a result, Ni_3_S_2_-based catalysts have been the most extensively studied. The HER performance of recently reported Ni_3_S_2_-based HER catalysts is summarized in Table 4.

### 3.4. Ni_3_S_4_-Based Catalysts

**Figure 8:**
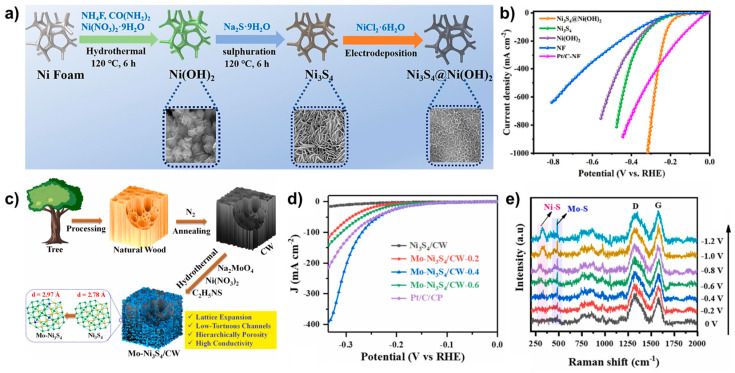
(**a**) Schematic illustration of the synthesis of Ni_3_S_4_/Ni(OH)_2_. (**b**) LSV curves comparing of Ni_3_S_4_/Ni(OH)_2_ with control samples in 1.0 M KOH. (**c**) Schematic representation of the synthesis of Mo–Ni_3_S_4_/CW. (**d**) LSV curves comparing Mo–Ni_3_S_4_/CW with control groups in 1.0 M KOH. (**e**) In situ Raman spectra of Mo-Ni_3_S_4_/CW within the HER potential range. Reproduced with permission from Elsevier [86,87].

Ni_3_S_4_@Ni(OH)_2_ catalyst was synthesized via a two-step hydrothermal method followed by electrodeposition (Figure 8a) [86]. The two-step hydrothermal process resulted in the formation of Ni_3_S_4_ on the Ni foam, followed by electrodeposition in an electrolyte containing 0.7 M NiCl_2_·6H_2_O to form Ni_3_S_4_@Ni(OH)_2_. SEM images revealed a morphology of interlocked nanosheets stacked together. The introduction of Ni(OH)_2_ onto Ni_3_S_4_ transformed its hydrophobic surface into a hydrophilic one, reducing the contact angle to nearly 0°. Ni_3_S_4_@Ni(OH)_2_ catalyst exhibited excellent HER activity with η_100_ of 212.6 mV in 1.0 M KOH, significantly outperforming single-phase Ni_3_S_4_ (η_100_ = 312.4 mV) and Ni(OH)_2_ (η_100_ = 350.4 mV) (Figure 8b). Furthermore, Ni_3_S_4_@Ni(OH)_2_ was employed as both anode and cathode materials in an anion exchange membrane water electrolyzer (AEMWE) system. The Ni_3_S_4_@Ni(OH)_2_|| Ni_3_S_4_@Ni(OH)_2_ setup exhibited superior performance, achieving a cell potential of 1.84 V at 500 mA cm^−2^, surpassing the noble metal-based IrO_2_||Pt/C catalyst system at high current densities. During 100 h of operation at 500 mA cm^−2^, the Ni_3_S_4_||Ni_3_S_4_ configuration showed a 3.7% potential increase, whereas Ni_3_S_4_@Ni(OH)_2_|| Ni_3_S_4_@Ni(OH)_2_ exhibited only a 1.9% increase, indicating that the introduction of Ni(OH)_2_ significantly enhanced the stability of Ni_3_S_4_. 

Shi et al. synthesized a molybdenum-doped Ni_3_S_4_ catalyst grown on carbonized wood (Mo–Ni_3_S_4_/CW) via a hydrothermal process (Figure 8c) [87]. SEM images revealed that the CW exhibited a porous structure with microchannel walls, which enhanced the interaction between the electrolyte and the catalyst material. The Mo–Ni_3_S_4_ formed rough nanosheets on the CW, contributing to its excellent catalytic activity with overpotential of η_10_ = 17 mV and η_100_ = 270 mV in 1.0 M KOH. Mo doping resulted in superior activity compared to Ni_3_S_4_/CW (η_10_ = 280 mV) and Pt/C (η_10_ = 96 mV and η_100_ = 284 mV) (Figure 8d). In-situ Raman spectroscopy showed an increase in the intensity of Ni–S and Mo–S bands upon an applied potential, indicating that Mo partially substituted Ni and intercalated into Ni_3_S_4_, expanding the lattice distance and enhancing electronic interactions (Figure 8e). DFT calculations indicated that the water dissociation energy and hydrogen adsorption Gibbs free energy at the Mo sites in Mo–Ni_3_S_4_ were close to zero, the lowest values among the compared sites, suggesting that Mo not only improved the intrinsic activity of the Ni sites in Ni_3_S_4_ but also played a critical role in the overall catalytic performance enhancement.

Ge et al. synthesized Ni_3_S_4_–MoS_2_ using a one-step hydrothermal method [88]. The SEM images showed that Ni_3_S_4_–MoS_2_ formed hierarchical nanospheres composed of nanosheets, with the heterostructure resulting in larger and thinner nanosheets than MoS_2_. This structural advantage provided Ni_3_S_4_-MoS_2_ with superior HER performance, achieving η_10_ = 116 mV in 1.0 M KOH, compared to single-phase MoS_2_ (η_10_ = 235 mV) and Ni_3_S_4_ (η_10_ = 318 mV). DFT calculations were performed to evaluate the chemisorption free energies of OH (Δ*E*_OH_) and H (Δ*E*_H_), revealing that Ni_3_S_4_ had a lower Δ*E*_OH_, whereas MoS_2_ had a lower Δ*E*_H_, indicating a preference for OH chemisorption on Ni_3_S_4_ and H chemisorption on MoS_2_ in the heterostructure. Furthermore, Ni_3_S_4_–MoS_2_ demonstrated a lower free energy barrier for water dissociation than either single-component Ni_3_S_4_ or MoS_2_, highlighting the improved HER performance of the hetrostructure. The HER performance of recently reported Ni_3_S_4_-based catalysts is summarized in Table 5.

### 3.5. Ni_9_S_8_-Based Catalysts

**Figure 9:**
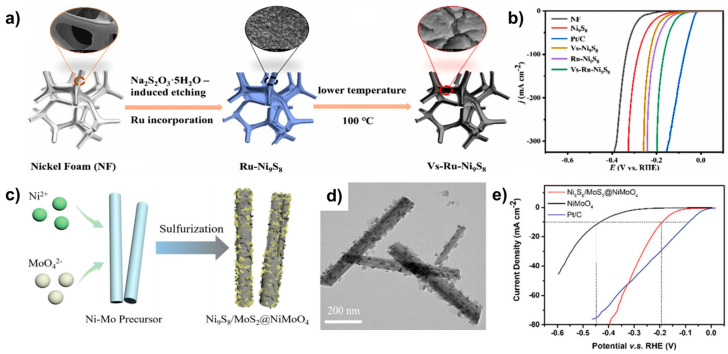
(**a**) Schematic illustration of the synthesis of Vs–Ru–Ni_9_S_8_. (**b**) LSV curves comparing of Vs–Ru–Ni_9_S_8_ and its comparison group in 1.0 M KOH. (**c**) Schematic representation of the synthesis of Ni_9_S_8_/MoS_2_@NiMoO_4_. (**d**) TEM image of Ni_9_S_8_/MoS_2_@NiMoO_4_. (**e**) LSV curves comparing of Ni_9_S_8_/MoS_2_@NiMoO_4_ and control samples in 1.0 M KOH. Reproduced with permission from Elsevier [91] and Wiley [92].

Gao et al. synthesized Ru-doped Ni_9_S_8_ with S vacancies (Vs–Ru–Ni_9_S_8_) using a hydrothermal process followed by low-temperature annealing at 100 °C (Figure 9a) [91]. SEM images revealed that Vs–Ru–Ni_9_S_8_ exhibited a layered rock-like morphology, while TEM images confirmed its 2D nanosheet structure. Electron paramagnetic resonance (EPR) spectral measurements revealed that Vs–Ru–Ni_9_S_8_, unlike Ru–Ni_9_S_8_, displayed a strong symmetric EPR signal at g = 2.003, indicating the successful formation of S vacancies. In 1.0 M KOH, Vs–Ru–Ni_9_S_8_ demonstrated superior HER activity with η_10_ = 94 mV compared to Ru-Ni_9_S_8_ (η_10_ =123 mV) (Figure 9b). The presence of S vacancies facilitated rapid ion transfer and exposed more active sites, contributing to the enhanced catalytic performance. 

Chen et al. synthesized Ni_9_S_8_/MoS_2_@NiMoO_4_ through a hydrothermal process followed by annealing under an Ar atmosphere (Figure 9c) [92]. SEM images revealed that Ni_9_S_8_/MoS_2_@NiMoO_4_ formed nanorods with a porous structure, unlike the smooth surface of NiMoO_4_. TEM images confirmed that 1D NiMoO_4_ nanorods formed the core, whereas the Ni_9_S_8_/MoS_2_ nanosheets constituted the shell (Figure 9d). These structural characteristics, which exposed more active sites and provided rapid charge transfer pathways, led to Ni_9_S_8_/MoS_2_@NiMoO_4_ exhibiting excellent HER activity with η_10_ = 190 mV in 1.0 M KOH and significantly outperforming single-phase NiMoO_4_ (η_10_ = 434 mV) (Figure 9e). The HER performance of recently reported Ni_9_S_8_-based HER catalysts is summarized in Table 6.

### 3.6. Heterostructured Catalysts Between Nickel Sulfides

**Figure 10:**
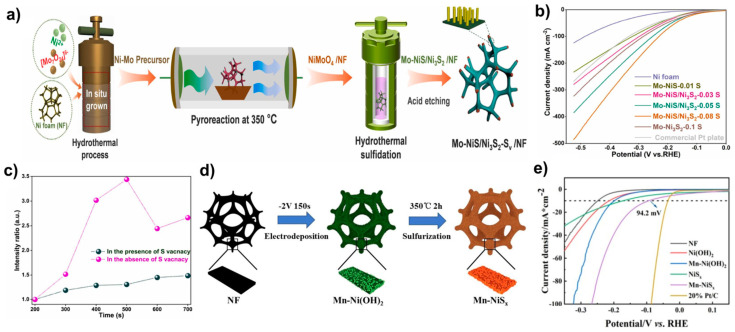
(**a**) Schematic illustration of the synthesis of Mo–NiS/Ni_3_S_2_–S_v_/NF. (**b**) LSV curves of Mo-NiS/Ni_3_S_2_/NF compared with control samples in 1.0 M KOH. (**c**) S–H stretching vibration intensity ratio from in situ Raman spectra comparing Mo–NiS/Ni_3_S_2_-free S_v_/NF and Mo–NiS/Ni_3_S_2_-rich S_v_/NF. (**d**) Schematic representation of the synthesis of Mn–NiS/Mn–Ni_3_S_4_. (**e**) LSV curves of Mn–NiS/Mn–Ni_3_S_4_ and its comparison group in 1.0 M KOH. Reproduced with permission from Elsevier [93,94].

Zhang et al. prepared Mo-doped NiS/Ni_3_S_2_ on Ni foam containing S vacancies (Mo–NiS/Ni_3_S_2_–Sv/NF), as illustrated in Figure 10a [93]. The synthesis involved a hydrothermal method to produce a Ni-Mo precursor, followed by thermal treatment under an Ar atmosphere to obtain NiMoO_4_/NF. Subsequent hydrothermal treatment with a sulfur source yielded Mo–NiS/Ni_3_S_2_/NF (denoted as Mo–NiS/Ni_3_S_2_–0.08S). To introduce S vacancies (S_v_), the material was etched in HCl, and the vacancy amount was controlled by varying the etching times (0, 30, 60, and 90 min), resulting in samples named Mo–NiS/Ni_3_S_2_-free S_v_, Mo–NiS/Ni_3_S_2_-poor S_v_, Mo–NiS/Ni_3_S_2_-rich S_v_, and Mo–NiS/Ni_3_S_2_-excess S_v_. SEM images confirmed the formation of nanorod-aggregated spheres in Mo–NiS/Ni_3_S_2_/NF. The heterostructured Mo–NiS/Ni_3_S_2_ catalyst demonstrated enhanced HER activity with η_100_ of 167 mV in 1.0 M KOH, outperforming Mo–NiS (η_100_ = 322 mV) and Mo–Ni_3_S_2_ (η_100_ = 283 mV) (Figure 10b). The catalysts with S vacancies exhibited superior HER activity compared to S-free catalysts, with the Mo–NiS/Ni_3_S_2_-rich S_v_ sample (etched for 60 min) achieving a remarkable performance of 230 mV at 100 mA cm^−2^ without iR compensation. In situ Raman spectra indicated that the S–H band of Ni_3_S_2_ appeared at a lower potential when S vacancies were present, indicating that S vacancies facilitate the formation of the S–H intermediate more easily, thereby enhancing HER activity. Furthermore, the intensity of the S–H band over reaction time showed that in the presence of S vacancies, the intensity gradually increased, whereas in the absence of S vacancies, the intensity increased abruptly (Figure 10c). This behavior suggests that S vacancies prevent the accumulation of S-H on the catalyst surface. This allows the proton, generated from water dissociation at the Ni site of Mo-NiS, to interact properly with Ni_3_S_2_ and enhance catalytic performance.

The Mn–NiS/Mn–Ni_3_S_4_ (denoted as Mn-NiS_x_) catalyst was prepared by electrochemical deposition followed by annealing (Figure 10d) [94]. Initially, Mn–Ni(OH)_2_ was synthesized via electrodeposition, and subsequent vulcanization with sulfur powder under an Ar atmosphere produced Mn–NiS/Mn–Ni_3_S_4_. SEM images revealed that the disordered lamellar spheres of Mn–Ni(OH)_2_ transformed into a well-ordered arrangement of nano-micro spheres with a specific pattern, indicating that Mn doping and vulcanization contributed to the regular morphology, unlike the disordered structure observed without Mn doping. This ordered morphology, induced by Mn doping and vulcanization, created surface areas highly favorable for HER. Mn–NiS/Mn–Ni_3_S_4_ demonstrated superior activity with an overpotential of η_10_ = 94.2 mV and η_100_ = 267 mV in 1.0 M KOH, outperforming irregular Mn–Ni(OH)_2_ (η_10_ = 197 mV and η_100_ = 373 mV) and NiS/Ni_3_S_4_ (η_10_ = 172 mV and η_100_ = 407 mV) (Figure 10e).

**Figure 11:**
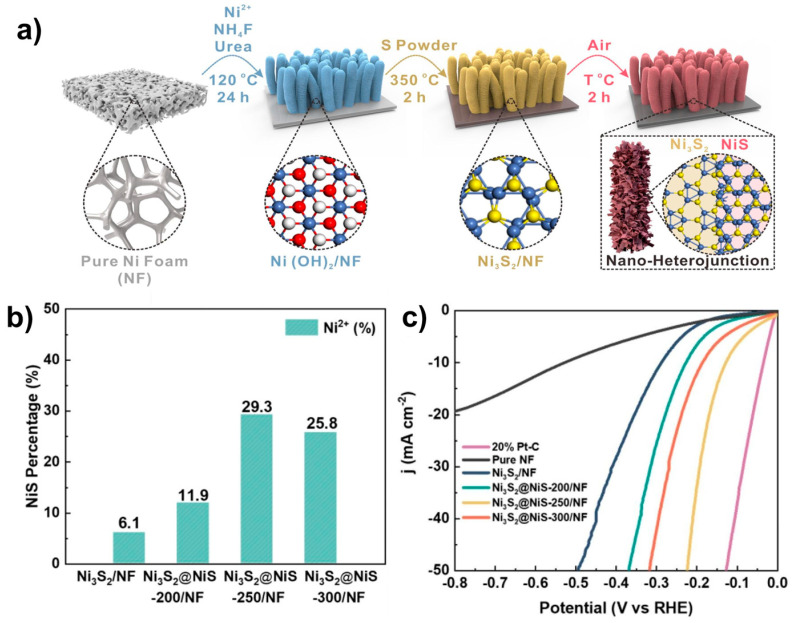
(**a**) Schematic representation of the synthesis of NiS@Ni_3_S_2_/NF. (**b**) XPS analysis of surface Ni^2+^ percentage of Ni_3_S_2_/NF and NiS@Ni_3_S_2_/NF samples annealed at 200, 250, and 300 °C. (**c**) LSV curves comparing of NiS@Ni_3_S_2_/NF and control samples in 1.0 M KOH. Reproduced with permission from Elsevier [95].

Chen et al. synthesized Ni_3_S_2_@NiS through a hydrothermal process followed by a two-step annealing process (Figure 11a) [95]. First, Ni(OH)_2_ nanowires were synthesized via the hydrothermal method followed by annealing with sulfur powder to form Ni_3_S_2_. The nanowires were then annealed in air at 200, 250, and 300 °C to yield Ni_3_S_2_@NiS, denoted as Ni_3_S_2_@NiS–200/NF, Ni_3_S_2_@NiS–250/NF, and Ni_3_S_2_@NiS–300/N, respectively. SEM images showed a uniform wire-like morphology, with nanowires forming an interconnected 3D network. XPS spectra confirmed the presence of Ni^2+^ and Ni^3+^ in both Ni_3_S_2_/NF and Ni_3_S_2_@NiS, with the proportion of Ni^2+^ increasing with higher calcination temperatures. Ni_3_S_2_@NiS–250/NF exhibited the highest Ni^2+^ proportion, indicating the highest NiS content, which correlated with enhanced HER performance (Figure 11b). Ni_3_S_2_@NiS–250/NF exhibited the best HER activity with η_10_ = 129 mV in 1.0 M KOH, with all heterostructure catalysts outperforming single-phase Ni_3_S_2_ (η_10_ = 298 mV), as shown in Figure 11c. DFT calculations revealed that Ni_3_S_2_@NiS has a hydrogen adsorption free energy (ΔG_H*_) closer to zero compared to single-phase NiS and Ni_3_S_2_, indicating that the heterostructure effectively optimizes hydrogen binding energy, enhancing the catalytic performance. The HER performance of recently reported heterostructured catalysts between nickel sulfides is summarized in Table 7. 

## 4. Summary and Perspective 

To achieve truly sustainable net-zero carbon emissions, hydrogen production must transition to clean green hydrogen production, which generates no greenhouse gas emissions. For widespread implementation of water electrolysis systems, efficient catalysts are essential for both cathodic HER and anodic OER. Currently, the typical catalysts are precious Pt-based materials, underscoring the urgent need for highly active and stable non-precious alternatives. Nickel sulfide is a promising candidate for HER because of its low cost, abundance, stability in electrolytes, and excellent electrical conductivity. Various nickel sulfide catalysts, including NiS, NiS_2_, Ni_3_S_2_, Ni_3_S_4_, and Ni_8_S_9_, have been explored as HER catalysts; however, their single-phase forms often exhibit low activity and poor stability, limiting their ability to replace noble metal-based catalysts. To address this issue, strategies such as heteroatom doping, heterostructure construction, and vacancy engineering have been employed. This review summarizes the recent research progress of nickel sulfide-based catalysts for HER, categorized by stoichiometry, along with their synthetic methods, characterization techniques, and impacts on HER performance. 

For the further development of nickel sulfide-based catalysts, several considerations should be considered. First, a more systematic approach is needed for nickel sulfide catalysts. Ni_3_S_2_-based catalysts have been predominantly studied, while NiS, NiS_2_, Ni_3_S_4_, and Ni_8_S_9_ have received less attention. To enhance HER activity, nickel sulfide catalysts are often combined with other components, such as metals, metal sulfides, metal oxides, and metal hydroxides, to form heterostructure catalysts. However, the intrinsic properties of stoichiometric nickel sulfides and the effects of these additional components on HER performance are poorly understood. Therefore, systematic investigations into the interactions between stoichiometric nickel sulfides and other components are crucial for improving the activity and stability of nickel sulfide-based catalysts. 

Second, the identification of active sites and the mechanisms behind the enhanced HER activity of nickel sulfide-based catalysts requires further exploration via using in situ characterization techniques. Although many studies have focused primarily on HER performance, understanding active sites and activity origins has often relied heavily on computational simulations. Given that most strategies involve heteroatom doping, heterostructure construction, and vacancy engineering, there is an urgent need for experimental evidence regarding the nature of real active sites and their origins. Advanced in situ characterizations, such as in situ Raman spectroscopy or in situ X-ray absorption spectroscopy (XAS), can provide valuable insights into the active sites and activity mechanism. A combined approach using in situ characterization techniques and computational simulations can significantly advance the development of nickel sulfide-based catalysts for HER.

Lastly, for industrial applications, improvements in HER performance and synthesis methods are necessary. Few reports have indicated that nickel sulfide-based catalysts exhibit superior activity compared with typical noble metal-based catalysts, particularly when operating at high current densities (e.g., ~ Acm^−2^). Moreover, limited studies have investigated the performance of nickel sulfide-based catalysts in single-cell systems. To assess the feasibility of nickel sulfide-based catalysts for water electrolysis systems, it is essential to demonstrate their operation at high current densities at the single-cell level. Additionally, economical synthesis methods are critical for mass production. Current synthesis processes often involve multistep thermal treatments, which limit the scalability and cost-effectiveness. Therefore, more straightforward synthesis methods are needed, along with processes that eliminate the need for additional post-synthesis treatments of the catalyst.

Water electrolysis is a promising method for hydrogen production, and the development of highly active and stable non-precious catalysts is essential for widespread adoption. Although various nickel sulfide-based catalysts have been studied for HER, further advancements are needed to improve their performance for practical applications. By combining systematic investigations into the interactions between stoichiometric nickel sulfides and other components with in situ characterizations, researchers can gain insights into the active sites and HER mechanisms, ultimately leading to the design of more efficient nickel sulfide-based catalysts.

## Figures and Tables

**Table 1:** HER performances of hexagonal NiS-based catalysts.

Catalyst	Substrate	Electrolyte	Synthesis Method	Overpotential		Ref
				η_10_	η_100_	
NiS/NiSe_2_	GC ^1)^	1.0 M KOH	Hydrothermal	155 mV	-	[20]
Mo_2_N/NiS	CFP ^2)^	1.0 M KOH	Calcination and Hydrothermal	254 mV	403 mV *	[21]
NC ^3)^/NiS–CeO_2_	NF ^4)^	1.0 M KOH	Hydrothermal	47 mV	139 mV *	[22]
MoS_2_/NiS	NF	1.0 M KOH	Hydrothermal	87 mV	189 mV *	[23]
α–NiS@NDCS ^5)^	NF	1.0 M KOH	Hydrothermal	173 mV	-	[24]
NiS@CoNi_2_S_4_/NC ^6)^	NF	1.0 M KOH	Calcination	126 mV	210 mV *	[25]
MoS_2_/rGO/NiS	GC	1.0 M KOH	Hydrothermal	169 mV	301 mV *	[26]
NiS@MoS_2_	NF	1.0 M KOH	Calcination and Hydrothermal	146 mV	-	[27]

^1)^ Glassy carbon. ^2)^ Carbon fiber paper. ^3)^ N-doped carbon coated. ^4)^ Nickel foam. ^5)^ N-doped carbon dots. ^6)^ N-doped carbon. Data marked with * are estimated from the LSV.

**Table 2:** HER performance of the rhombohedral NiS-based catalysts.

Catalyst	Substrate	Electrolyte	Synthesis Method	Overpotential		Ref
				η_10_	η_100_	
NiS/MoS_2_	CC ^1)^	1.0 M KOH	Electrodeposition and Hydrothermal	18 mV	93 mV	[28]
ReS_2_/NiS	NF	1.0 M KOH	Hydrothermal	78 mV	181 mV *	[29]
NiS/MoS_2_	CP ^2)^	1.0 M KOH	Hydrothermal	119 mV	314 mV *	[30]
MoS_2_/NiS	NF	1.0 M KOH	Hydrothermal	84 mV	168 mV *	[31]
Zn–NiS	NF	1.0 M KOH	Hydrothermal	208 mV	-	[32]
NiFeCr–S–NiS	NF	1.0 M KOH	Calcination and Hydrothermal	131 mV	254 mV *	[33]

^1)^ Carbon cloth. ^2)^ Carbon paper. Data marked with * are estimated from the LSV.

**Table 3:** HER performance of NiS_2_-based catalysts.

Catalyst	Substrate	Electrolyte	Synthesis Method	Overpotential		Ref
				η_10_	η_100_	
Co–NiS_2_–CeO_2_	NF	1.0 M KOH	Calcination and Hydrothermal	88 mV	213 mV *	[34]
NiS_2_/MoS_2_/CNTs	GC	1.0 M KOH	Hydrothermal	149 mV	320 mV *	[35]
NiS_2_–Ni_3_C@C	GC	1.0 M KOH	Calcination and Hydrothermal	78 mV	189 mV *	[36]
V–NiS_2_	CC	1.0 M KOH	Calcination and Hydrothermal	85 mV	247 mV *	[37]
RuO_2_/NiS_2_	NF	1.0 M KOH	Hydrothermal	71 mV	-	[38]
VO_2_–NiS_2_	CC	1.0 M KOH	Hydrothermal	96 mV	225 mV *	[39]
Zr–MOF/NiS_2_	NF	1.0 M KOH	Calcination	72 mV	-	[40]
(Fe, Ni)S_2_@MoS_2_/NiS_2_	NF	1.0 M KOH	Calcination and Hydrothermal	91 mV	225 mV *	[41]
Co–NiS_2_/MoS_2_	CFP	1.0 M KOH	Calcination	89 mV	166 mV	[42]
MoO_x_@NiS_2_	NF	1.0 M KOH	Calcination and Hydrothermal	101 mV	323 mV *	[43]
MoS_2_/NiS_2_	CC	1.0 M KOH	Hydrothermal	80 mV	175 mV	[44]
P–NiS_2_	NF	1.0 M KOH	Calcination and Solvothermal	73 mV	173 mV *	[45]

Data marked with * are estimated from the LSV.

**Table 4:** HER performance of Ni_3_S_2_-based catalysts.

Catalyst	Substrate	Electrolyte	Synthesis Method	Overpotential		Ref
				η_10_	η_100_	
Co–MoS_2_/Ni_3_S_2_	NF	1.0 M KOH	Hydrothermal	43 mV	201 mV	[46]
Ni_3_S_2_@NiCoN	NF	1.0 M KOH	Calcination and Hydrothermal	63 mV	174 mV	[47]
A–MoS_2_/Ni_3_S_2_^1)^	NF	1.0 M KOH	Hydrothermal	95 mV	191 mV	[48]
Li, V–Ni_3_S_2_	NF	1.0 M KOH	Hydrothermal	90 mV	183 mV	[49]
Ni_3_S_2_/MoS_2_	CC	1.0 M KOH	Hydrothermal	105 mV *	189 mV	[50]
VS_4_/Ni_3_S_2_	NF	1.0 M KOH	Solvothermal	140 mV	268 mV	[51]
Co_2_P–Ni_3_S_2_	NF	1.0 M KOH	Calcination and Hydrothermal	-	110 mV	[52]
1T–MoS_2_/Ni_3_S_2_/LDH	NF	1.0 M KOH	Electrodeposition and Hydrothermal	104 mV	342 mV *	[55]
W/Mo–Ni_3_S_2_	NF	1.0 M KOH	Hydrothermal	136 mV	271 mV *	[56]
S–NiMoO_4_/Ni_3_S_2_	NF	1.0 M KOH	Electrodeposition and Hydrothermal	107 mV	244 mV *	[57]
CoS_1.097_/Ni_3_S_2_	NF	1.0 M KOH	Hydrothermal	74 mV	-	[58]
MoO_2_/Ni_3_S_2_	NF	1.0 M KOH	Hydrothermal	74 mV	200 mV *	[59]
Mo_5_N_6_/Ni_3_S_2_	NF	1.0 M KOH	Calcination and Hydrothermal	59 mV	313 mV *	[60]
CoS/Ni_x_P_y_/Fe–Ni_3_S_2_	NF	1.0 M KOH	Calcination and Hydrothermal	49 mV	203 mV	[61]
Fe–Ni_3_S_2_	NF	1.0 M KOH	Electrodeposition	-	98 mV	[62]
Fe–MoS_2_/Ni_3_S_2_	NF	1.0 M KOH	Hydrothermal	74 mV	235 mV	[63]
Ni_3_S_2_/NiMoS	NF	1.0 M KOH	Electrodeposition and Hydrothermal	197 mV	197 mV	[64]
CoMoP–Ni_3_S_2_	NF	1.0 M KOH	Calcination and Hydrothermal	97 mV	192 mV *	[65]
Fe–Ni_3_S_2_/Ni_2_P	NF	1.0 M KOH	Calcination and Hydrothermal	112 mV	198 mV	[66]
FeOOH/Ni_3_S_2_	NF	1.0 M KOH	Electrodeposition	92 mV	232 mV *	[67]
La–Ni_3_S_2_/MoS_2_	NF	1.0 M KOH	Hydrothermal	-	154 mV	[68]
Ni_3_S_2_@MoS_2_@Ni_3_Si_2_	NF	1.0 M KOH	Hydrothermal	84 mV	143 mV *	[69]
NiWO_4_–Ni_3_S_2_@NiO	NF	1.0 M KOH	Electrodeposition and Hydrothermal	89 mV	210 mV *	[70]
Ni(OH)_x_/Ni_3_S_2_	NF	1.0 M KOH	Electrochemical activation and Hydrothermal	54 mV	126 mV	[71]
Ru–Ni_3_S_2_/Ni_x_P_y_	NF	1.0 M KOH	Calcination and Hydrothermal	51 mV	126 mV *	[72]
FeWO_4_–Ni_3_S_2_@C ^2)^	NF	1.0 M KOH	Calcination and Solvothermal	50 mV	173 mV *	[73]
Au–Ni_3_S_2_	NF	1.0 M KOH	Hydrothermal	97 mV	188 mV *	[74]
MoS_x_@Co_9_S_8_@Ni_3_S_2_	NF	1.0 M KOH	Hydrothermal	77 mV	180 mV *	[75]
Co–NiOOH/Ni_3_S_2_	NF	1.0 M KOH	Electrodeposition and Hydrothermal	87 mV	203 mV	[76]
NiO/Ni_3_S_2_	CC	1.0 M KOH	Electrodeposition	91 mV	210 mV *	[77]
Ni_3_S_2_/Ni(OH)_2_	NF	1.0 M KOH	Electrodeposition	66 mV	312 mV *	[78]
(Ni_3_S_2_–MoS_2_)/TiO_2_	NF	1.0 M KOH	ALD ^3)^ and Hydrothermal	49 mV	118 mV *	[79]
Ni_3_S_2_–MoS_2_	NF	1.0 M KOH	Solvothermal	103 mV	207 mV *	[80]
MoS_2_–Ni_3_S_2_	NF	1.0 M KOH	Hydrothermal and Calcination	109 mV	214 mV *	[81]
Co_3_O_4_@Mo–Co_3_S_4_–Ni_3_S_2_	NF	1.0 M KOH	Hydrothermal	116 mV	214 mV *	[82]
Co–Ni_3_S_2_	NF	1.0 M KOH	ALD and Hydrothermal	62 mV	160 mV *	[83]
Co–Ni_3_S_2_	NF	1.0 M KOH	Calcination and Hydrothermal	102 mV (η_20_)	158 mV	[84]
Mo–Ni_3_S_2_	NF	1.0 M KOH	Hydrothermal	90 mV	176 mV *	[85]

^1)^ Amorphous. ^2)^ Carbon encapsulated. ^3)^ Atomic layer deposition. Data marked with * are estimated from the LSV.

**Table 5:** HER performance of Ni_3_S_4_-based catalysts.

Catalyst	Substrate	Electrolyte	Synthesis Method	Overpotential		Ref
				η_10_	η_100_	
Ni_3_S_4_@Ni(OH)_2_	NF	1.0 M KOH	Electrodeposition and Hydrothermal	118 mV	213 mV	[86]
Mo–Ni_3_S_4_	CW ^1)^	1.0 M KOH	Hydrothermal	17 mV	270 mV	[87]
Ni_3_S_4_–MoS_2_	NF	1.0 M KOH	Hydrothermal	116 mV	-	[88]
Ni_3_S_4_@MoS_2_	CC	1.0 M KOH	Hydrothermal	97 mV	174 mV	[89]
Ni_3_S_4_/Ni/Ni(OH)_2_	TM ^2)^	1.0 M KOH	Electrodeposition	54 mV	185 mV *	[90]

^1)^ Carbonized wood. ^2)^ Ti mesh. Data marked with * are estimated from the LSV.

**Table 6:** HER performance of Ni_9_S_8_-based catalysts.

Catalyst	Substrate	Electrolyte	Synthesis Method	Overpotential		Ref
				η_10_	η_100_	
Vs–Ru–Ni_9_S_8_	NF	1.0 M KOH	Hydrothermal	94 mV	170 mV *	[91]
Ni_9_S_8_/MoS_2_@NiMoO_4_	NF	1.0 M KOH	Calcination and Hydrothermal	190 mV	-	[92]

Data marked with * are estimated from the LSV.

**Table 7:** HER performance of heterostructured catalysts between nickel sulfides.

Catalyst	Substrate	Electrolyte	Synthesis Method	Overpotential		Ref
				η_10_	η_100_	
Mo–NiS/Ni_3_S_2_	NF	1.0 M KOH	Calcination and Hydrothermal	-	167 mV	[93]
Mn–NiS/Mn–Ni_3_S_4_	NF	1.0 M KOH	Calcination and Electrodeposition	94 mV	267 mV	[94]
Ni_3_S_2_@NiS	NF	1.0 M KOH	Calcination and Hydrothermal	129 mV	-	[95]
NiS/Ni_3_S_4_	GC	1.0 M KOH	Calcination and Hydrothermal	263 mV	495 mV *	[96]
Mo–Ni_9_S_8_/Ni_3_S_2_	NF	1.0 M KOH	Hydrothermal	116 mV	240 mV	[97]
Ni_3_S_4_/NiS_2_/FeS_2_	NF	1.0 M KOH	Hydrothermal	196 mV	-	[98]
NiS–NiS_2_–Ni_3_S_4_	NF	1.0 M KOH	Calcination and Hydrothermal	68 mV	-	[99]
P–Ni_3_S_2_–NiS	NF	1.0 M KOH	Hydrothermal	141 mV	376 mV *	[100]
V–Ni_3_S_2_–NiS	NF	1.0 M KOH	Hydrothermal	85 mV	218 mV	[101]

Data marked with * are estimated from the LSV.

## Data Availability

No new data were created or analyzed in this study. Data sharing is not applicable to this article.
